# Fabrication of Biomimetic Bone Tissue Using Mesenchymal Stem Cell-Derived Three-Dimensional Constructs Incorporating Endothelial Cells

**DOI:** 10.1371/journal.pone.0129266

**Published:** 2015-06-05

**Authors:** Jun-Ichi Sasaki, Masanori Hashimoto, Satoshi Yamaguchi, Yoshihiro Itoh, Itsumi Yoshimoto, Takuya Matsumoto, Satoshi Imazato

**Affiliations:** 1 Department of Biomaterials Science, Osaka University Graduate School of Dentistry, Osaka, Japan; 2 Department of Restorative Dentistry and Endodontology, Osaka University Graduate School of Dentistry, Osaka, Japan; 3 Department of Biomaterials, Okayama University, Okayama, Japan; Georgia Regents University, UNITED STATES

## Abstract

The development of technologies to promote vascularization of engineered tissue would drive major developments in tissue engineering and regenerative medicine. Recently, we succeeded in fabricating three-dimensional (3D) cell constructs composed of mesenchymal stem cells (MSCs). However, the majority of cells within the constructs underwent necrosis due to a lack of nutrients and oxygen. We hypothesized that incorporation of vascular endothelial cells would improve the cell survival rate and aid in the fabrication of biomimetic bone tissues *in vitro*. The purpose of this study was to assess the impact of endothelial cells combined with the MSC constructs (MSC/HUVEC constructs) during short- and long-term culture. When human umbilical vein endothelial cells (HUVECs) were incorporated into the cell constructs, cell viability and growth factor production were increased after 7 days. Furthermore, HUVECs were observed to proliferate and self-organize into reticulate porous structures by interacting with the MSCs. After long-term culture, MSC/HUVEC constructs formed abundant mineralized matrices compared with those composed of MSCs alone. Transmission electron microscopy and qualitative analysis revealed that the mineralized matrices comprised porous cancellous bone-like tissues. These results demonstrate that highly biomimetic bone tissue can be fabricated *in vitro* by 3D MSC constructs incorporated with HUVECs.

## Introduction

Approaches that attempt to fabricate living tissues *ex vivo* are increasing with the development of tissue engineering technologies and biomaterials [1, 2]. *In vitro* tissue engineering techniques are considered to be applicable not only to regenerative medicine but also to drug-discovery technology and histogenetics research [3–6]. Recently, methods for the generation of several kinds of tissues have been developed [1, 7, 8]. Three-dimensional (3D) bone-like constructs were formed by seeding human-derived mesenchymal stem cells (hMSC) into collagen-hydroxyapatite scaffolds [9] and bioactive glass ceramics [10]. Also, liver-like constructs were fabricated by culturing hepatocytes with cellulose and hyaluronan-gelatin hydrogels [11]. Furthermore, other groups have reported cell-sheet engineering and constructed myocardium-like tissues by accumulating membranous cell aggregates [12, 13].

3D biomaterial scaffolds are frequently used in tissue engineering to support cell proliferation and determine a specific shape. However, there are many concerns about the use of scaffolds, including: 1) it is difficult to control the absorption rate to precisely match the rate of new tissue formation [14]; and 2) the remaining material or degradation byproducts occasionally hamper tissue regeneration [15]. Therefore, scaffold-free approaches could further progress tissue-engineered medical products.

We previously reported that scaffold-free 3D cell constructs could be fabricated using a thermo-responsive hydrogel that alters its volume depending on the surrounding temperature [16]. Furthermore, we have also shown that bone-like tissue and cartilage tissue were formed in the process of endochondral ossification by osteogenic induction of mouse-derived MSCs [17]. The cell constructs consisted solely of cells without a scaffold, hence they hold great promise as a novel bone graft material. However, the majority of cells within the cell construct were necrotized by insufficient oxygen and nutrient supply. Thereby, small and immature mineralized matrices were formed within these cell constructs.

We hypothesized that the survival of the inner cells could be improved by incorporating human vascular endothelial cells (HUVECs) into the cell constructs, resulting in efficient biomimetic bone fabrication *in vitro*. If developed, this technology could help towards the realization of *in vitro* tissue engineering [18].

The purpose of this study was to assess the influence of HUVECs incorporated into hMSC-derived cell constructs (MSC/HUVEC constructs) during short- and long-term culture, and fabricate biomimetic bone tissue *in vitro* by inducing their osteogenic differentiation.

## Materials and Methods

### Cell culture

Human mesenchymal stem cells (hMSCs; Riken, Tsukuba, Japan) were cultured in Dulbecco’s Modified Eagle’s Medium (DMEM) containing 20% fetal bovine serum (FBS). Human umbilical vein endothelial cells (HUVECs; Riken) were cultured in Endothelial Cell Basal Medium-2 supplemented with SingleQuots (EBM-2; Lonza, Walkersville, MD). hMSC and HUVECs were maintained in a humidified incubator at 37°C with 5% CO_2_.

To assess cell proliferation in osteogenic differentiation medium (dif-MEM), each cell type was cultured in DMEM containing 20% FBS, beta-glycerophosphate disodium salt hydrate (1 × 10^-2^ mol/l, Sigma-Aldrich, St. Louis, MO), ascorbic acid (50 μg/ml, Sigma-Aldrich), dexamethasone (1 × 10^-6^ mol/l, Sigma-Aldrich), and 10 mM of calcium chloride solution for controlling the calcium concentration in the medium. Each cell type was seeded into 24-well cell culture plates (1.0 × 10^4^ cells), and counted by using a hemocytometer on days 1, 3, 7, and 12.

### Preparation of hMSC/HUVEC 3D constructs

The cell constructs were fabricated using a thermo-responsive poly(N-isopropylacrylamide) (poly-NIPAAm) hydrogel mold [16]. Briefly, a 3D UV curable polymer for poly-NIPAAm gel molding was designed using graphic modeling software (Freeform, Geomagic, Rock Hill, SC) and manufactured with a 3D printing system (Eden, Objet, Israel). A NIPAAm solution with polyethylene glycol dimethacrylate as the cross-linking reagent was poured into the chamber and refrigerated for 8 h. The polymerized hydrogel was created with holes (φ = 1.5 mm) that enabled cell spheroid formation.

Suspensions of hMSCs containing HUVECs at a rate of 0, 1, 2, and 5% of the total cell number (1.0 × 10^5^ cells) were poured into the holes of the gel to fabricate cell constructs composed of hMSCs and HUVECs (99:1–95:5), and hMSCs alone (100:0). After 24 h, each cell construct was harvested by reducing the temperature from 37°C to room temperature for 15 min. The cell constructs were cultured in dif-MEM with shaking on a seesaw shaker at 0.13 Hz to prevent the constructs from adhering to the culture substrate.

The diameter of the cell construct was measured throughout the culture period using images taken by a CCD camera (DS-Fi2, Nikon, Tokyo, Japan) equipped with a stereoscopic microscope (SMZ745T, Nikon) (n = 8).

### Histological analysis

Cell constructs (100:0–95:5) were cultured for 7 days, fixed with 4% paraformaldehyde and embedded in paraffin, which was then cut into 5-μm thick sections. For histological evaluation, sections of the cell construct were stained with hematoxylin and an aqueous eosin Y solution. Cells were counted by means of a grid (300 × 200 μm) under a light microscope in 4 independent specimens. For immunofluorescent staining, the sections were deparaffinized, then incubated in phosphate buffered saline (PBS) containing 0.1% Triton-X and 1% bovine serum albumin for 20 min. After being washed twice, the sections were incubated with antibodies against CD31 (anti-CD31; 1/50, DAKO, Carpinteria, CA), vascular endothelial growth factor (anti-VEGF; 1/200, Merck Millipore, Billerica, MA), or hepatocyte growth factor (anti-HGF; 1/100, Santa Cruz Biotechnology, Santa Cruz, CA) for 40 min, then incubated with a secondary antibody conjugated with Alexa Fluor 488 (Invitrogen, Carlsbad, CA) for 40 min, followed by nuclear staining with Hoechst33342 (Invitrogen). The stained sections were observed using a fluorescence microscope with a CCD camera (TE2000; Coolsnap cf, Nikon).

### Electron microscopy observation

Transmission electron microscopy (TEM) and scanning electron microscopy (SEM) analyses were carried out on the hMSC only (100:0) and hMSC/HUVEC constructs (95:5) at 7 and 50 days of culture according to a previous report [19]. Briefly, cell constructs were fixed with 2.5% glutaraldehyde for 24 h. The 3D cell constructs were then post-fixed in 1% osmium tetraoxide for 2 h, and washed and dehydrated in graded concentrations of ethanol (40–100%). These cell constructs were infiltrated and embedded in Epoxy resin (EPON 812, TAAB, Berks, UK). For TEM observation, ultrathin sections (100 nm) were then cut using a diamond knife on a microtome (Sorvall MT-5000, Du Pont, CA). The sections were collected on copper grids and stained with saturated aqueous uranyl acetate, counter stained with 4% lead citrate and observed under TEM (H-7100, Hitachi, Tokyo, Japan). For SEM observation, harvested cell constructs were coated with gold and observed by SEM (JSM-6390, JEOL, Tokyo, Japan).

### *In silico* analyses of HUVEC reticulation

It was conceived that HUVECs in the 3D cell construct could survive and form reticulated structures by the influence of growth factors secreted by the hMSCs. To confirm this hypothesis, *in silico* analysis was performed to investigate the reticulation mechanisms of HUVECs using CompuCell3D simulation software [20]. The HUVEC model, which doubled in number and migrated every 100 Monte Carlo Steps (MCSs), was located at the mass of the hMSC models. Both cell models were represented as polygonal lattices. The MSCs model was configured to secrete VEGF at 50 pg/h [21], which would diffuse at a speed of 0.042 μm^2^/s [22]. The simulations were carried out under the condition that HUVECs proliferated and migrated with or without the influences of VEGF up to 600 MCSs.

### Alkaline phosphatase (ALP) activity measurement

ALP activity of cells forming the 3D construct was measured using the ALP quantitative analysis kit (Wako, Osaka, Japan) according to the manufacture’s instruction. Briefly, cell constructs (100:0–95:5) cultured for 7 or 14 days were washed with PBS, and dispersed by ultrasonic agitation in cell lysis buffer (RIPA buffer; Takara Bio, Otsu, Japan). 100 μl of p-nitrophenyl phosphate reaction mixture and a 20 μl aliquot of each supernatant were applied to each well (96-well plate). After the plate was incubated for 15 min at 37°C, 80 μl of stop solution was added to each well. The optical density was quantified at 450 nm with a microplate reader (Bio-Rad, Hercules, CA). The experiment was performed in triplicate for each group and repeated three times.

### Characterization of precipitated minerals in hMSC/HUVEC constructs

Sections (5-μm thick) of the hMSC/HUVEC constructs cultured for 50 days were prepared and von Kossa staining or Alcian blue staining were conducted. The stained areas of mineralized aggregates were measured using images from the CCD camera (DS-Fi2) equipped with a light microscope (ECLIPSE Ci; Nikon). Four sections from four different cell constructs were used in the quantitative analysis.

The hardness of the mineralized aggregate was measured for investigating osteogenic-maturation of the hMSC/HUVEC constructs. Briefly, hMSC/HUVEC constructs cultured for 50 days were embedded in paraffin and then cut to the core using a microtome. The consistencies of the fabricated tissues were evaluated for micro-Vickers hardness using a micro hardness testing machine (MicroWiZhard; Mitsutoyo, Kawasaki, Japan) (n = 5).

The amount of mineral deposition produced by hMSCs in the cell constructs was investigated using the methylxylenol blue (MXB) method (Calcium E-Test Wako; Wako). hMSC/HUVEC cell constructs (100:0–95:5) cultured for 50 days were washed with ultrapure water and dried using a dry-heat sterilizer (MOV-112S; Sanyo, Tokyo, Japan). Dried cell constructs were dissolved in 1 M HCl and mixed with MXB and monoethanolamine buffer. The calcium concentrations of the solutions were determined by measuring the absorbance at 595 nm (n = 4).

### Fourier-transform infrared (FT-IR) spectroscopy

FT-IR spectroscopy was carried out to investigate the composition changes of precipitated matrices by incorporating HUVECs into the hMSC constructs. Cell constructs composed of hMSCs only (100:0) and hMSCs/HUVECs (95:5) cultured for 50 days were washed with ultrapure water and dried using a dry-heat sterilizer (MOV-112S). The samples were then dispersed in a micronized potassium bromide powder using a pestle. The mixed sample powder was loaded on a sample cup as a flat loose powder bed with a flat surface formed by a microspatula. FT-IR spectra were obtained by powder diffuse reflectance using an FT-IR spectrophotometer (FTIR-8300; Shimadzu, Kyoto, Japan). A total of 50 scans were collected with a range of 1900 to 700 cm^-1^ at a resolution of 1 cm^-1^.

### Statistical analysis

One-way analysis of variance (ANOVA) with a Tukey’s or Dunnett’s post hoc test was used for comparisons of more than two groups. The Student’s t-test was used for comparisons of two groups. A significant difference was defined for values of *p* < 0.05 or < 0.01.

## Results

### Fabrication of hMSC/HUVEC constructs

**Fig 1:**
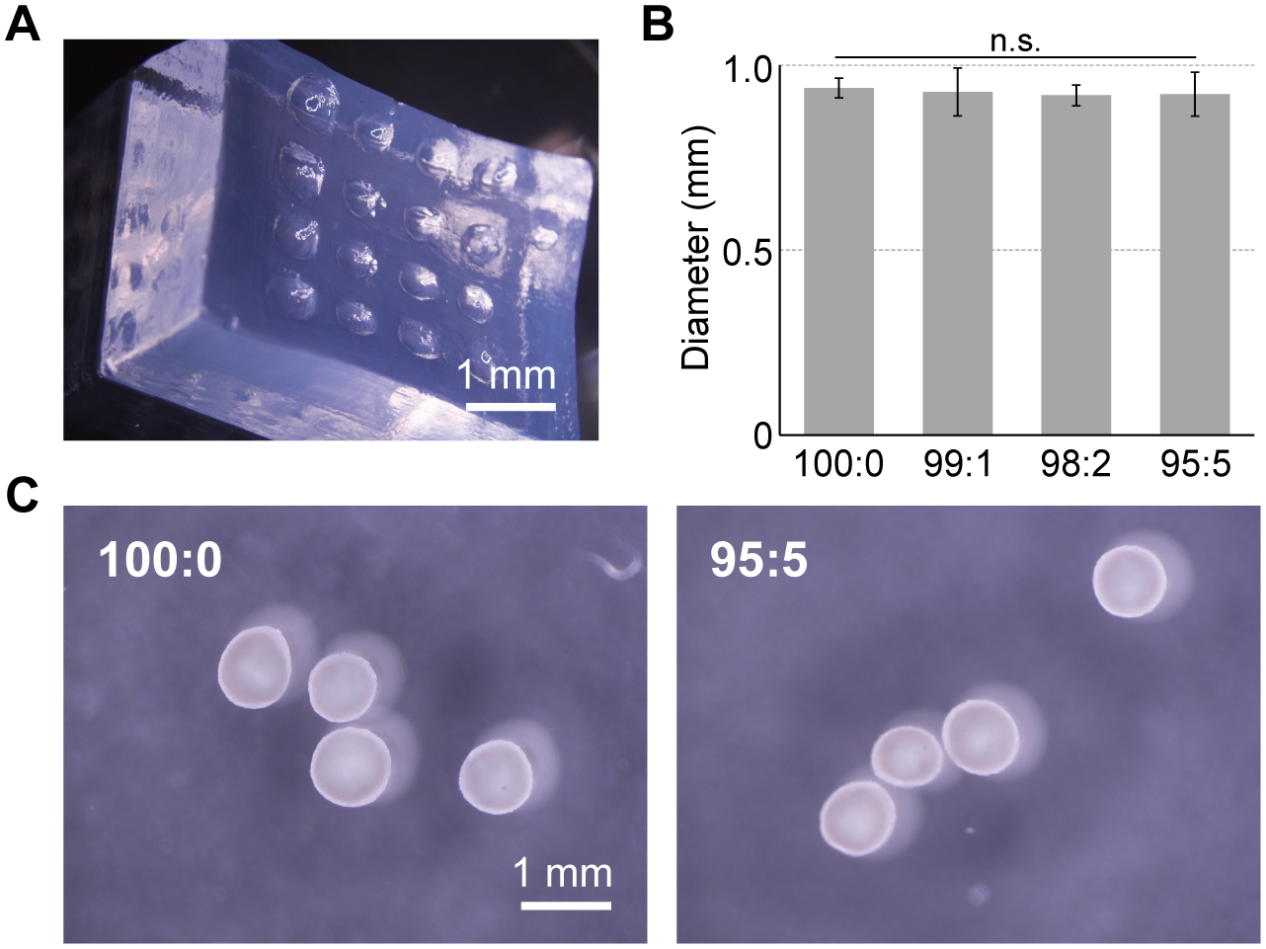
Fabrication of hMSC/HUVEC 3D cell constructs. Cell constructs were formed using a thermo-responsive poly-NIPAAm hydrogel (A) and were obtained as uniform sizes (B, C).

hMSC/HUVEC constructs were obtained by seeding hMSC and HUVEC suspensions into thermo-responsive poly-NIPAAm hydrogel (Fig 1). These cell constructs showed white color and uniform sizes (0.9–1.0 mm) at all ratios of HUVECs. However, the HUVEC only suspension did not form the globular shape (data not shown).

**Fig 2:**
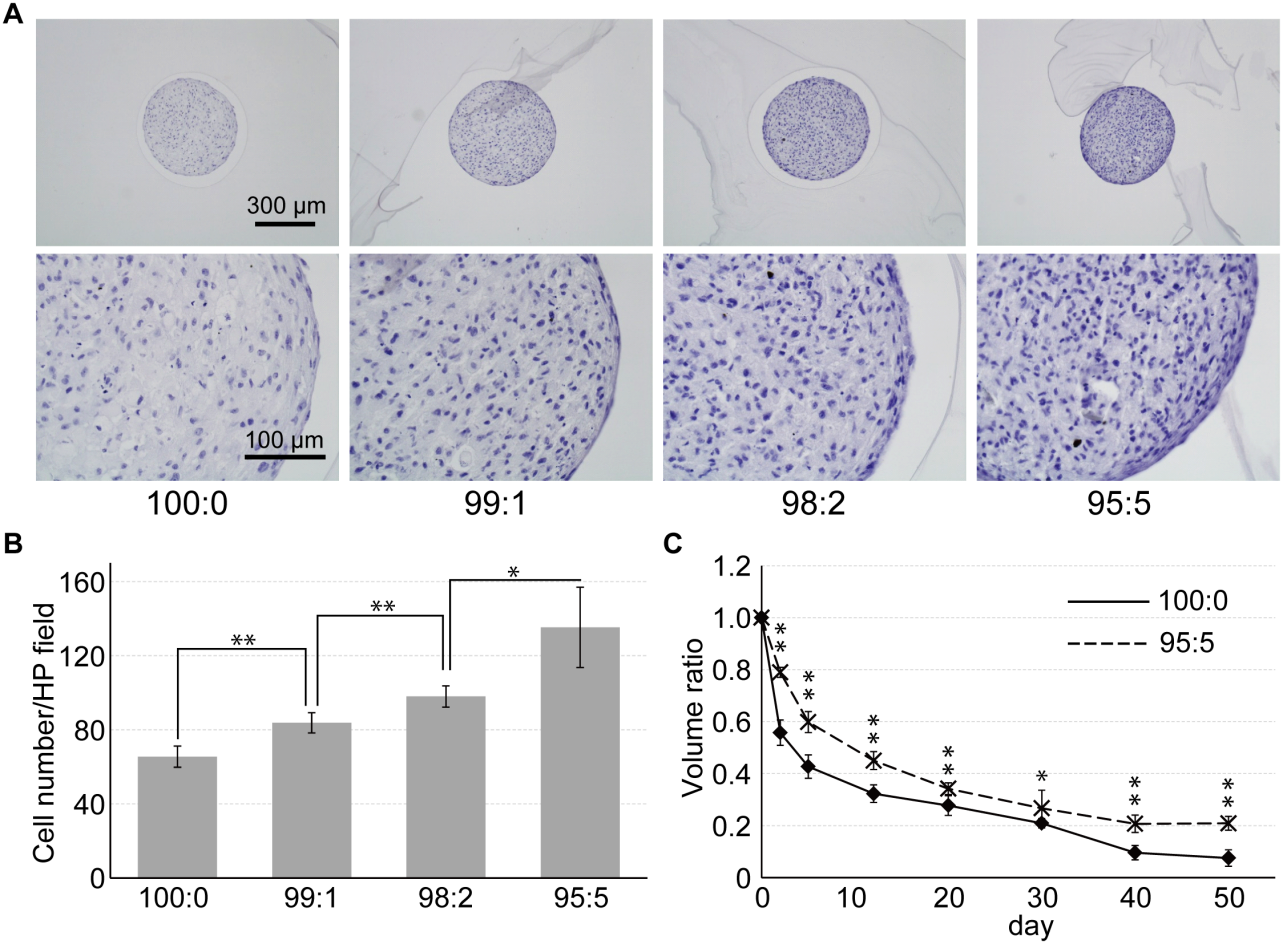
Growth of hMSC/HUVEC 3D cell constructs. (A) HE staining was carried out on each cell construct after 7 days in culture. (B) Cell numbers stained by hematoxylin were counted in 300 × 200 μm fields. Stained cells increased as the ratio of HUVECs increased. (C) As the size of the cell construct decreased, less size loss was found significantly in the 95:5 cell constructs throughout the culture period. (* *p* < 0.05; ** *p* < 0.01).

hMSC/HUVEC constructs were successfully cultured using the seesaw bioreactor. At day 7, HE stained images revealed that the number of stained cell increased as the ratio of HUVECs increased (Fig 2A and 2B). The size of the 100:0 cell constructs decreased during long-term culture, whereas less size loss was found in the 95:5 cell constructs over the culture period (Fig 2C).

### Internal structures of the 3D cell construct

**Fig 3:**
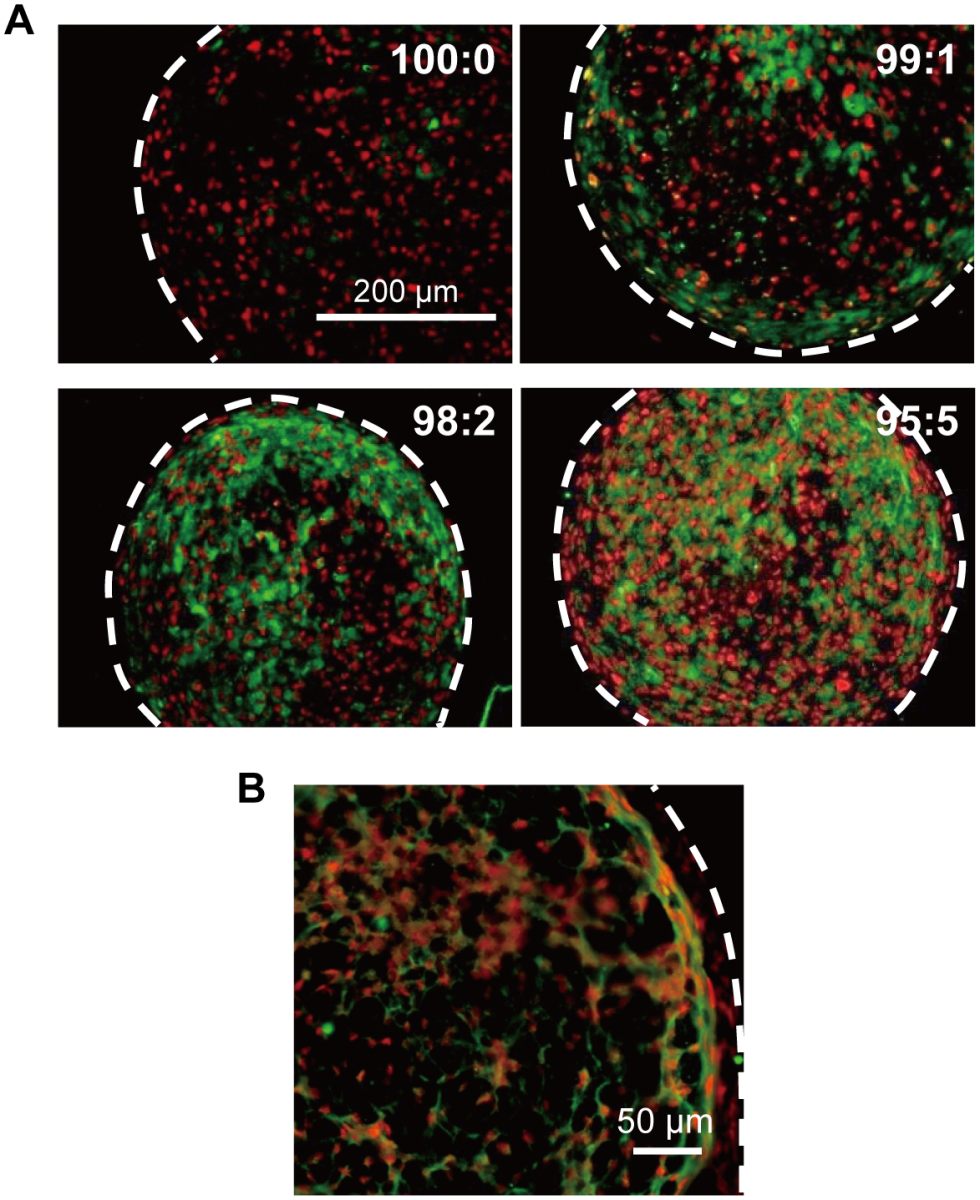
CD31 immunofluorescence stained images of hMSC/HUVEC 3D constructs. (A) Stained area of HUVECs (CD31-positive cells; green), which were more diffuse with the increasing ratio of HUVECs. The hMSCs only stained positive for Hoechst33342 (nucleus; red) (B) Representative magnified image of a hMSC/HUVEC (99:1) construct showing that HUVECs formed a reticulated structure within the 3D cell construct. White dotted lines indicate outline of cell construct.

HUVECs did not proliferate in dif-MEM and underwent necrosis within 1 week of 2D culture (S1 Fig). However, HUVECs were able to survive within the cell constructs. The number of CD31 positive cells increased incrementally with increasing HUVEC ratio in the cell constructs (Fig 3A). In addition, the magnified image revealed that hMSCs and HUVECs showed a particular distribution in the cell constructs. The hMSC/HUVEC construct was formed by 3–4 layers of hMSCs in the outermost region, 2–3 layers of HUVECs in the inside layer, and a reticulated structure of HUVECs in the nucleus of the cell construct (Fig 3B).

**Fig 4:**
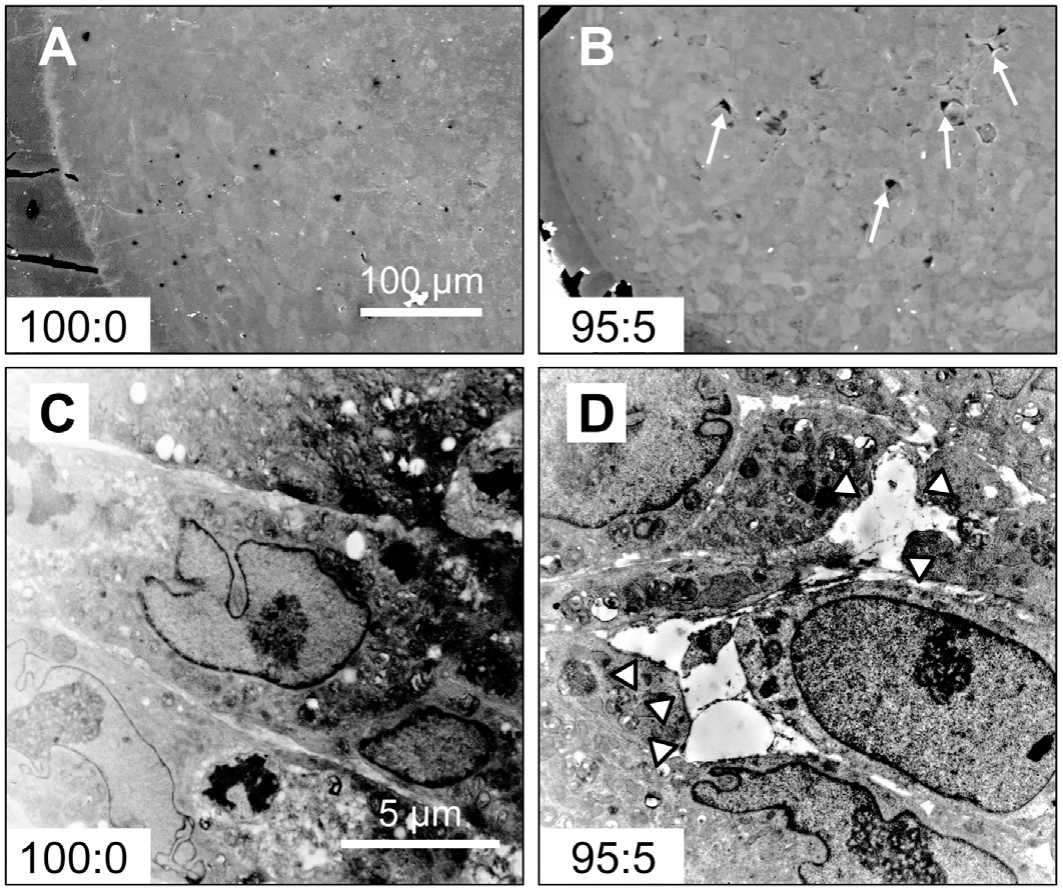
SEM and TEM images of 3D cell constructs. Cavities (arrows) were formed within the 95:5 cell constructs (B) but not in the 100:0 constructs (A). TEM images revealed that tight structures formed in the 100:0 cell constructs (C). By contrast, void spaces (arrowhead) between the cells were observed in 95:5 cell constructs (D).

SEM images showed that the 100:0 cell constructs were filled with cells (Fig 4A). However, the 95:5 samples exhibited some cell-free pores at 7 days of culture (Fig 4B). Detailed TEM analysis revealed that the MSCs were arranged densely in the 100:0 cell constructs (Fig 4C). By contrast, uneven gaps (maximum diameter; 5–10 μm) were generated between the cells in the 95:5 constructs (Fig 4D).

### Growth factors and differentiation of MSCs in 3D cell constructs

**Fig 5:**
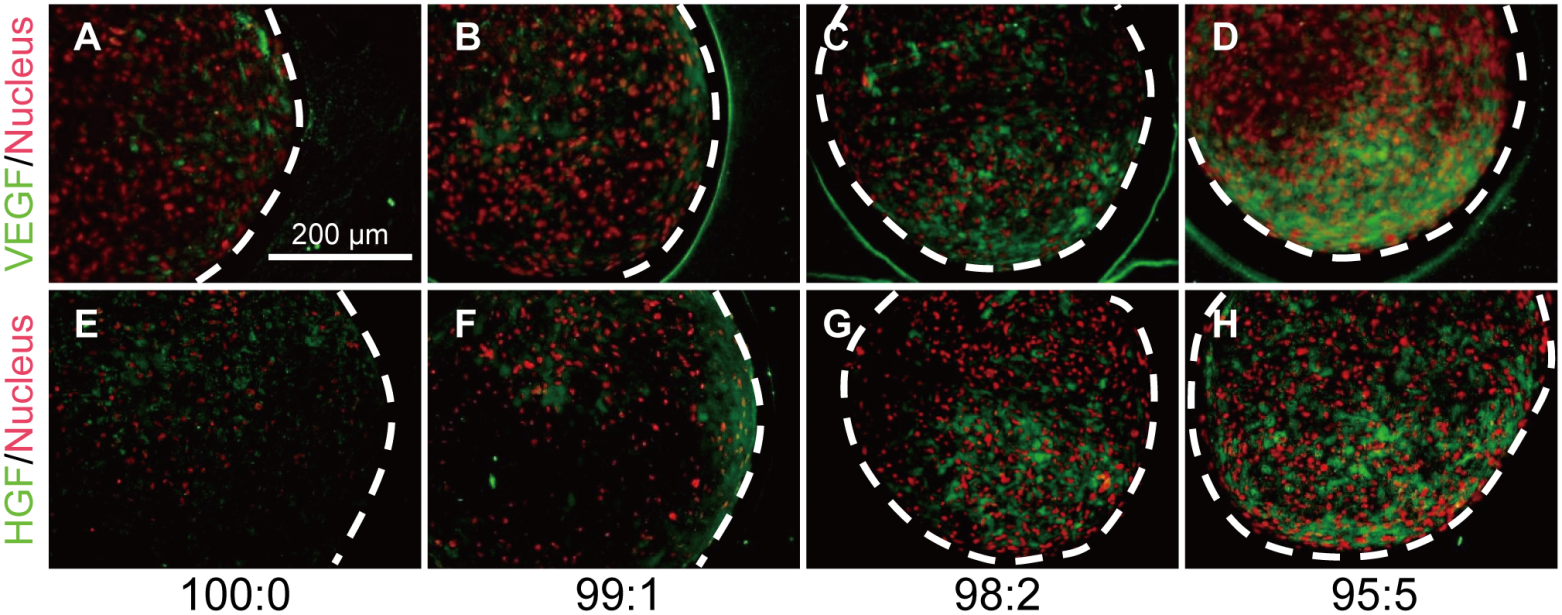
Immunofluorescence stained images of hMSC/HUVEC 3D constructs for angiogenic factors. (A–D) VEGF and (E–H) HGF were diffused within each cell construct. White dotted lines indicate outline of cell construct.

Immunofluorescence staining of angiogenic-related growth factors showed that co-culture with HUVECs expanded the areas positive for VEGF and HGF secreted by hMSCs (Fig 5).

**Fig 6:**
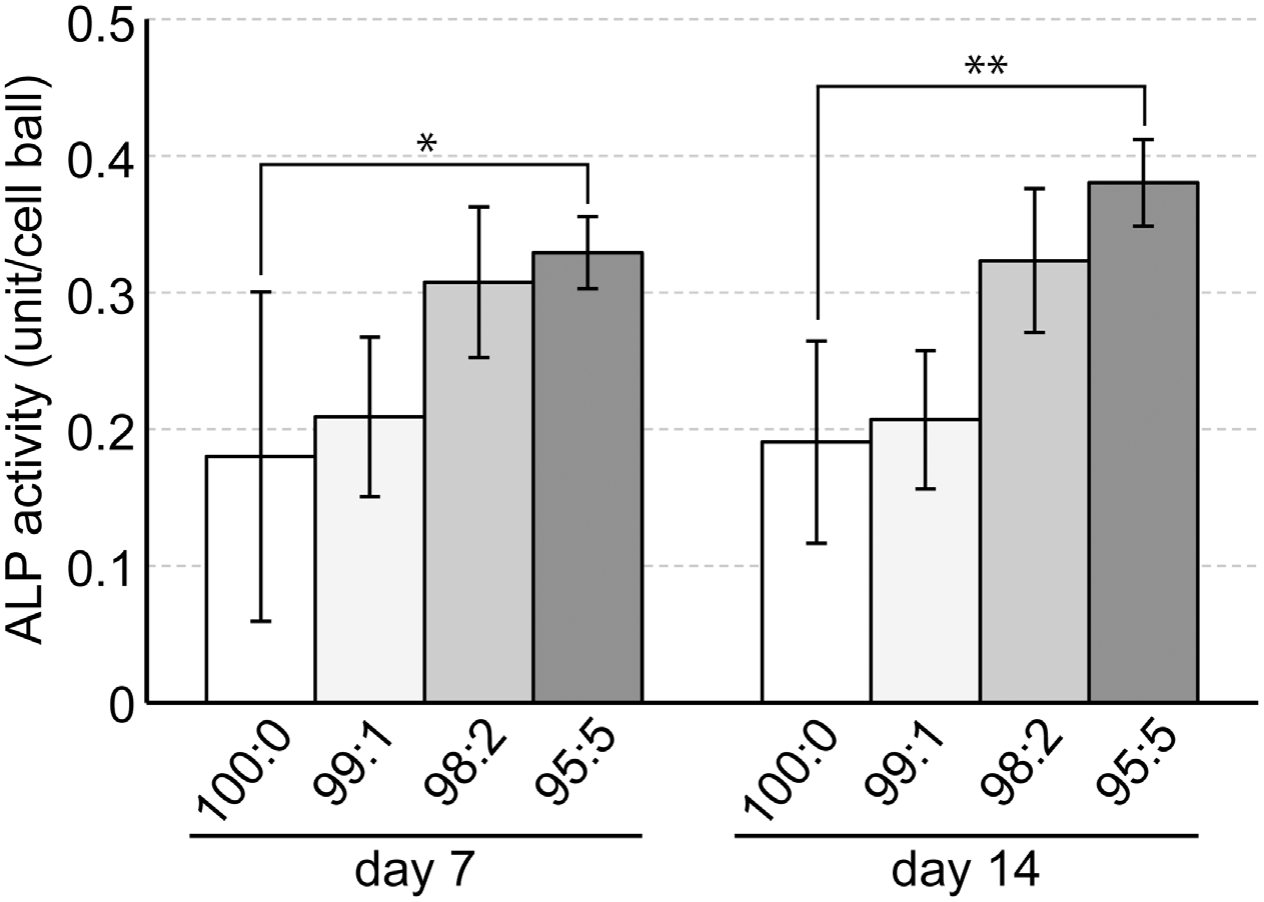
ALP activity of hMSC/HUVEC 3D constructs. HUVEC incorporated hMSC constructs (99:1–95:5) showed higher ALP activity compared with the hMSC only construct (100:0) at 7 and 14 days of culture (* *p* < 0.05; ** *p* < 0.01).

ALP activity in the 100:0 cell constructs at day 7 measured as 0.18 ± 0.12 units, whilst the 95:5 cell constructs had a significantly higher value of 0.33 ± 0.026 units. In addition, at day14, ALP activity in the 95:5 cell constructs measured as 0.38 ± 0.031 units, which was approximately two-fold the amount of the 100:0 cell constructs (0.19 ± 0.074 units) (Fig 6).

### *In silico* analyses

**Fig 7:**
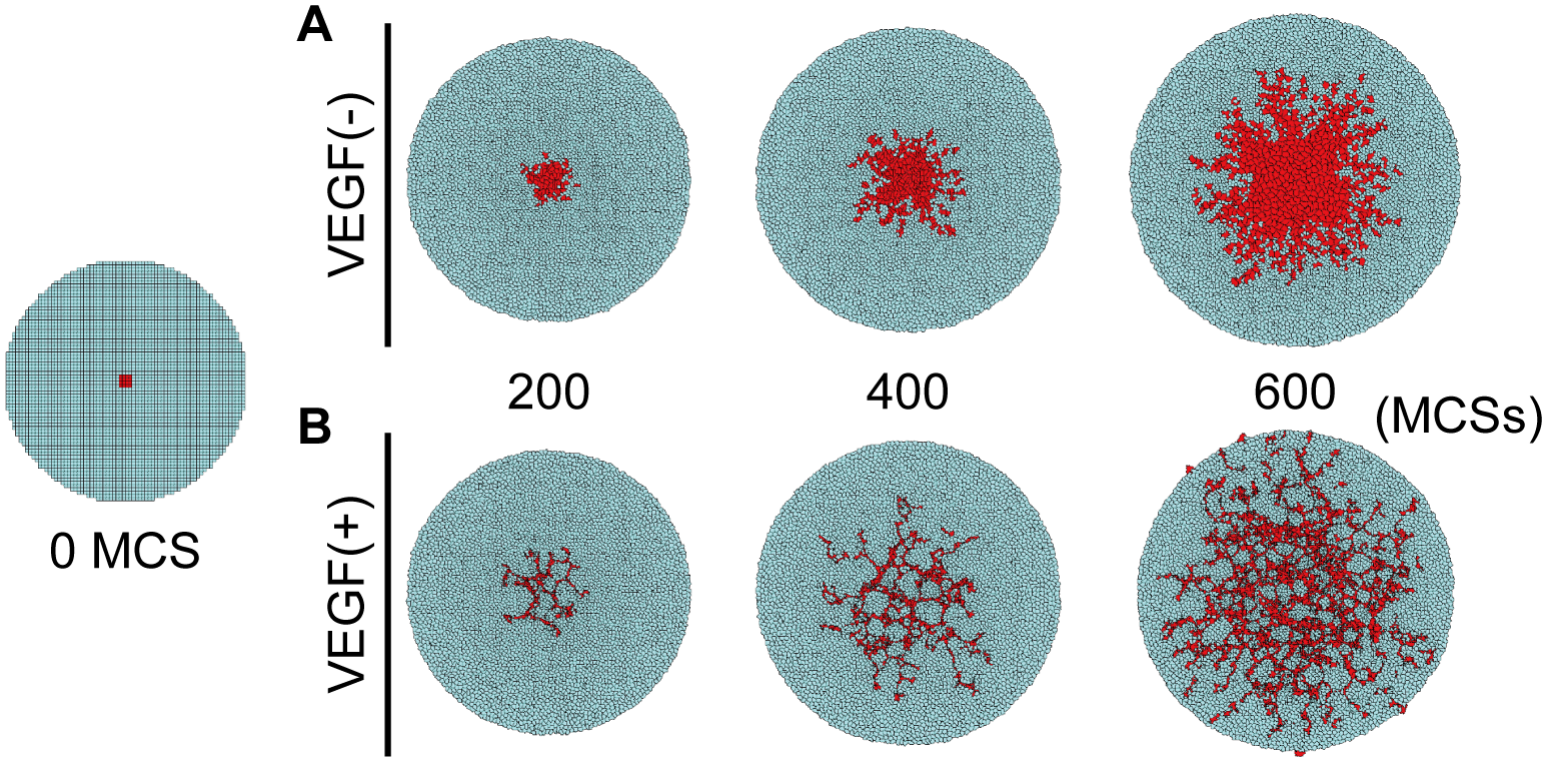
*In silico* analyses of the reticulate process of HUVECs. The HUVEC model (red polygons) was set in those of the hMSCs models (blue polygons) at 0 MCS. In absence of VEGF, HUVECs proliferated and colonized near their initial position (A). By contrast, in the presence of VEGF, HUVECs responded and spread across a wide area with the reticulate structure (B).

The results of the *in silico* analyses showed that when HUVECs were subjected to the influence of VEGF, HUVECs radiated outward after 200 MCSs (Fig 7B). When HUVECs were free of VEGF, HUVECs proliferated and colonized near their initial position (Fig 7A). At 600 MCSs, HUVECs that responded to VEGF spread across a wide area with a reticulate structure. In contrast, without the influence of VEGF, HUVECs created a greater cell mass but did not form a specific structure.

### Structures of mineralized matrices in cell constructs

**Fig 8:**
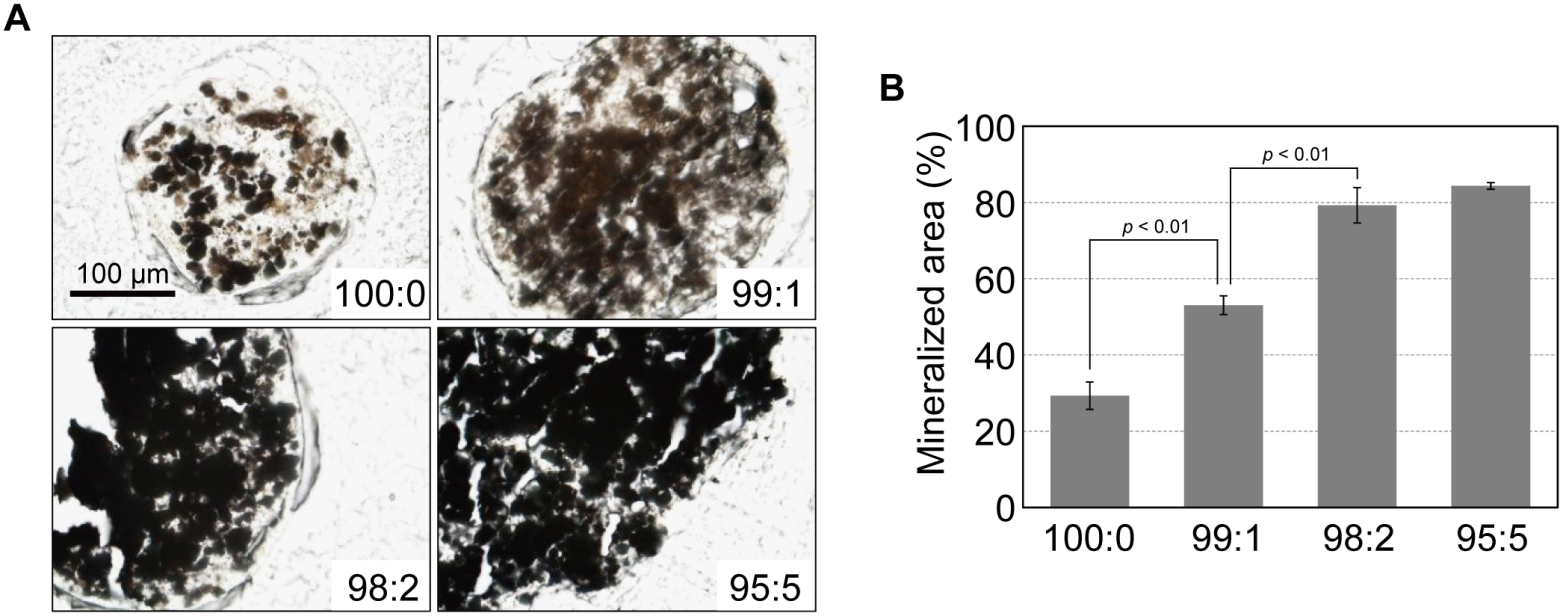
Mineral deposition in the hMSC/HUVEC 3D constructs. (A) Mineralized matrices within the cell constructs were detected by von Kossa staining as black aggregates, and (B) semi-quantitative assessment was performed (n = 4).

Alcian blue staining detected cartilage matrices within the cell constructs at day 50 of culture (S2 Fig). In addition, increased mineral deposition was observed in the hMSC/HUVEC constructs compared with the hMSC only constructs at day 50 of culture (Fig 8).

**Fig 9:**
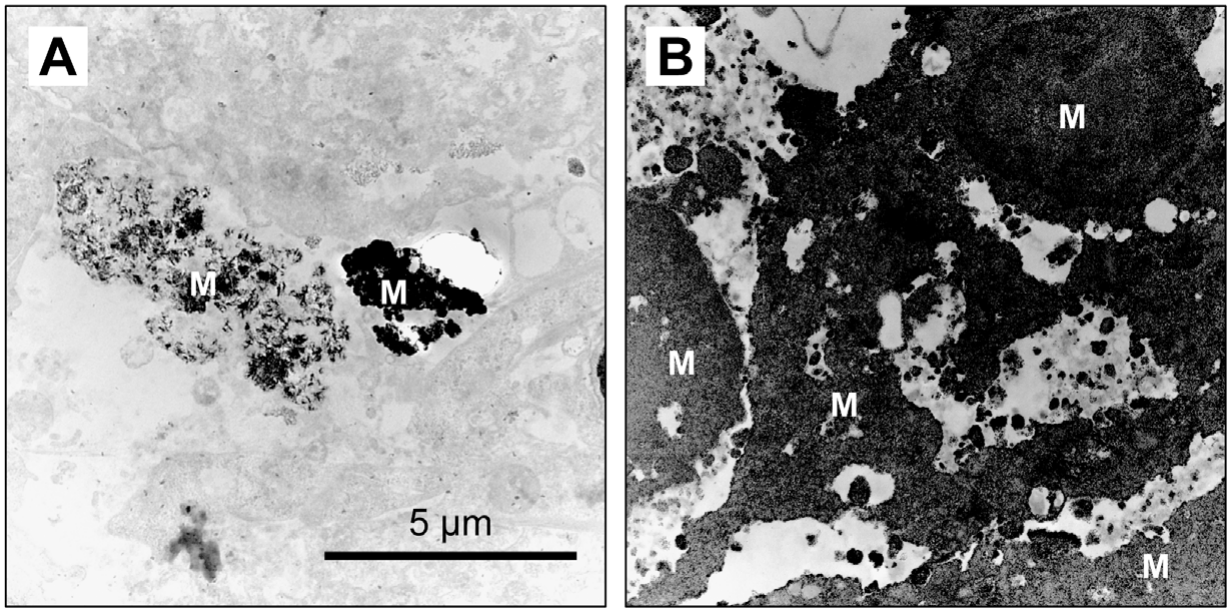
Self-organization of mineralized matrices in the 3D cell constructs after 50 days of culture. (A) Mineral deposition (M) was generated in the cytoplasm in the 100:0 cell constructs. (B) Mineral deposition resulted in a cancellous bone-like porous structure in the 95:5 cell constructs.

TEM images of the 100:0 cell constructs showed that some MSCs deposited calcified substances in the cytoplasm and extracellular matrix at day 50 of culture (Fig 9A). In the 95:5 cell constructs, the majority of cells were calcified completely and the mineralized matrices were connected with each other. In addition, these matrices were observed to build a porous structure inside the cell constructs comprising a cancellous bone-like structure (Fig 9B).

### Characterization of cell construct mineralized matrices

**Fig 10:**
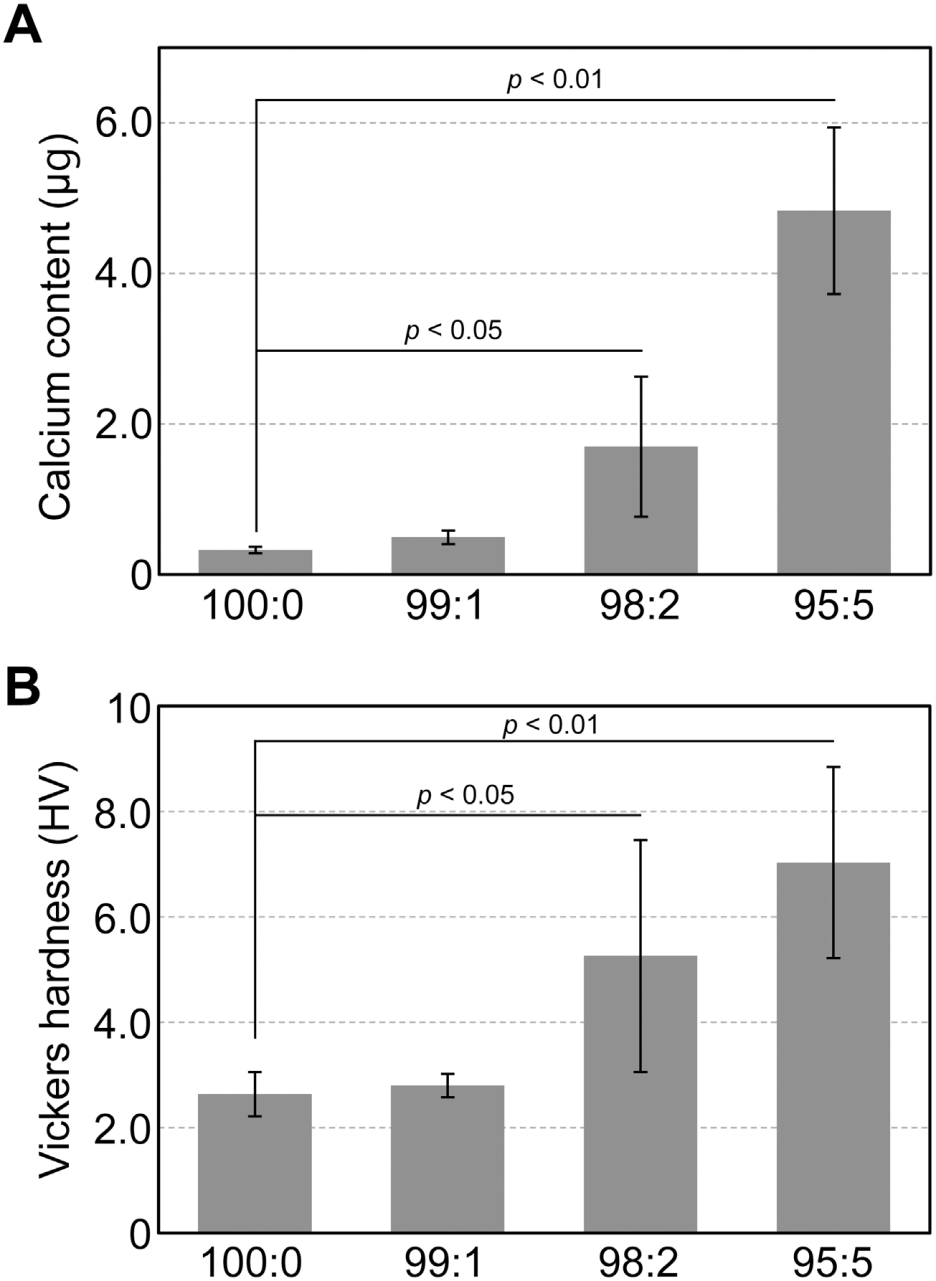
Characterization of mineralized matrices in hMSC/HUVEC 3D constructs. Mineralized matrices were evaluated by calcium content (A) and Vickers hardness (B). Both values increased following incorporation of HUVECs into the hMSC constructs.

Calcium content of the mineralized tissue was 0.32 ± 0.042 μg for the 100:0 cell constructs, which increased dramatically to 1.70 ± 0.93 μg for the 98:2 cell constructs and 4.83 ± 1.11 μg for the 95:5 cell constructs (Fig 10A). There were no significant differences in calcium contents between the 98:2 and 95:5 hMSC/HUVEC constructs. Fig 10B shows the results of the micro-Vickers hardness test of the hMSC/HUVEC constructs (100:0–95:5) cultured for 50 days. The cell constructs stiffened following incorporation of HUVECs, and their consistencies changed from 2.64 ± 0.41 HV for the 100:0 to 7.02 ± 1.81 HV for the 95:5 cell constructs.

**Fig 11:**
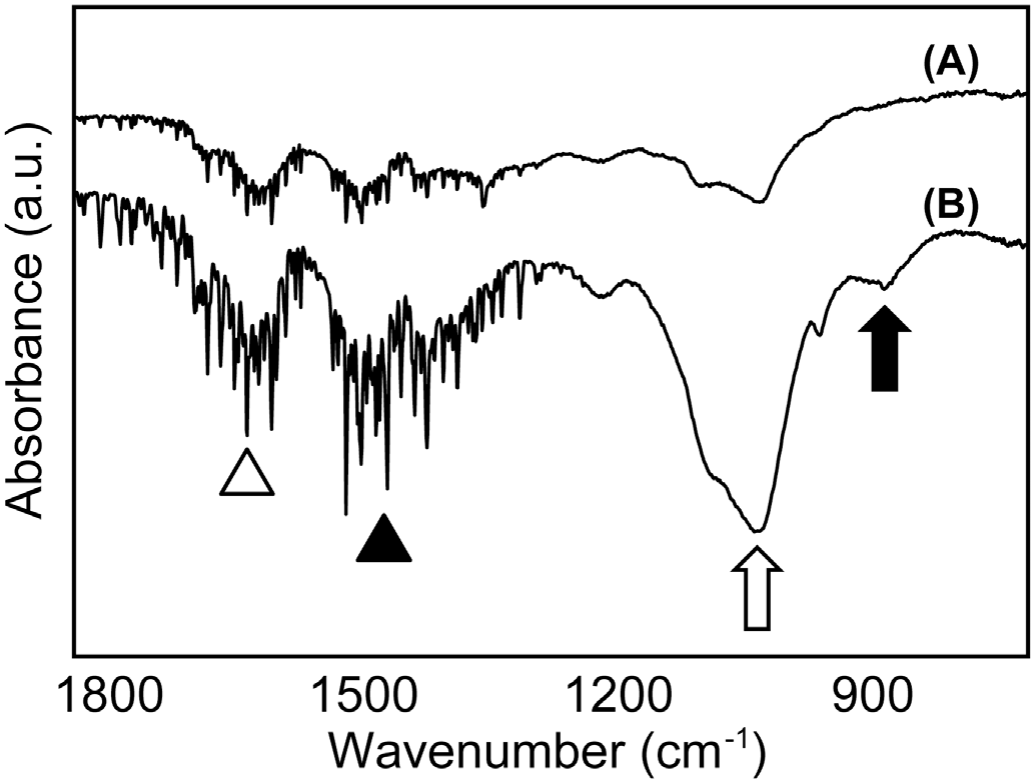
Comparisons of FT-IR spectra of (A) 100:0 and (B) 95:5 cell constructs. The IR spectra shows bands for amide I (1700–1630 cm^-1^, open arrowhead), amide II (1620–1520 cm^-1^, filled arrowhead), phosphate (1200–900 cm^-1^, open arrow), and carbonate (890–860 cm^-1^, filled arrow).

The IR absorption spectra of the cell constructs with and without HUVECs (95:5 and 100:0) are shown in Fig 11. The FT-IR spectroscopy analyses revealed that the hMSC cell constructs with HUVECs contained high bone-specific absorbance band vibrations of phosphate (1200–900 cm^-1^), carbonate (890–860 cm^-1^) and matrices rich in amino acids (amide I: 1700–1630 cm^-1^; amide II: 1620–1520 cm^-1^) [23]. hMSC constructs without HUVECs exhibited an undetectable absorbance band for carbonate, and the other bands were unclear, even after 50 days culture.

## Discussion

Despite significant progression in the science of *in vitro* tissue engineering, practical technologies are limited for cartilage regeneration [24, 25] and two dimensional tissues, for example the epidermis [26] and retina [27]. These matters are attributed to the lack of technologies that enable us to induce the structure of blood capillaries into the exogenously-produced tissues. Therefore, interior portions of fabricated tissues are necrotized over a short period due to the absence of a capillary network. Development of a technology that induces the capillary network into the fabricated tissues *in vitro* could provide a practical option that could be applicable for the fabrication of various tissues *in vitro* [18, 28, 29]. Some groups have reported the successful formation of vascular networks within tissue-like constructs or 3D scaffolds [30–34]. Zeitlin *et al*. [31] constructed an angiogenesis model by seeding dermal microvascular endothelial cells into a peptide hydrogel. Nishiguchi *et al*. [30] applied a sandwich culture based on the cell-accumulation technique, then fabricated highly dense tubular 3D networks.

We previously demonstrated that bone marrow-derived MSCs showed region-specific self-organization within the cell construct by prolonged culture with osteogenic differentiation medium [17]. In this study, we attempted to fabricate the capillary network in MSC 3D constructs by incorporating vein endothelial cells and to evaluate the occurrence of MSC self-organization within the construct.

Spherical cell constructs were fabricated by incorporating HUVECs at a ratio of 1%-5% with hMSCs; however HUVECs alone were unable to form cell aggregates. Cell–cell adhesion force is predominantly generated by neural (N)-cadherin in hMSCs and by vein endothelial (VE)-cadherin in HUVECs [35, 36]. Because our cell constructs were composed solely of cells (hMSCs and/or HUVECs), these results were considered to be caused by differences in cell–cell adhesion forces between the MSCs and HUVECs.

HUVECs, which were cultured using media containing angiogenic growth factors, necrotized following culture with osteogenic differentiation medium. In contrast, CD31 staining revealed that HUVECs survived within the hMSC constructs, even in the presence of osteogenic medium. In addition, our previous study reported that the centrally-located cells in the 3D construct were necrotized in a short culture period owing to the absence of oxygen and nutrition [16], however, the number of viable cells increased as the ratio of incorporated HUVECs increased. These results demonstrated that HUVECs were able to survive under some influence of the hMSCs. Additionally, less size loss was found in the 95:5 constructs compared with the 100:0 constructs. The relatively larger number of surviving cells in the 95:5 constructs likely secreted sufficient matrix molecules to maintain the 3D morphology of the cell constructs.

HUVECs and hMSCs showed specific distribution within the hMSC/HUVEC constructs, which contained cell layers of hMSCs in the outer region and a reticulate structure of HUVECs. In addition, cavities between the cells were observed by SEM and TEM. These results indicated that HUVECs self-organized under the influence of hMSCs and formed a luminal-like structure.

Because these results demonstrated that HUVECs were able to survive and self-organize under the influence of hMSCs, we next investigated the distribution of VEGF and HGF, two well-known angiogenic factors [37–39]. Accordingly, productions of VEGF and HGF by the hMSCs were promoted incrementally by the proportion of HUVECs. This result indicated that HUVECs could form a reticulate structure and survive within the 3D cell construct via the presence of these growth factors. We next carried out *in silico* analyses to confirm these findings. The self-organization process of HUVECs within the hMSC constructs was simulated using CompuCell3D software, which specializes in analyses of cell–cell interactions [20, 40]. These analyses demonstrated that single HUVECs could form the reticulate structure supported by growth factors secreted from the hMSCs.

It has recently been reported that the osteogenic differentiation of hMSCs is significantly enhanced following co-culture with HUVECs in 2D cultures [41–44], 3D cultures with scaffolds [45, 46], and also *in vivo* [47, 48]. These studies indicate that the extent of cell–cell communication between the hMSCs and HUVECs with secreted cytokines, such as bone morphogenetic proteins (BMPs), might be able to enhance the osteogenesis of hMSCs [41]. The results reported here, which correlate well with those of previous reports, revealed that crosstalk between HUVECs and hMSCs occurred within the 3D scaffold-free environment, and such paracrine interactions are likely valuable when fabricating biomimetic bone tissue *in vitro*.

Next, we compared the properties of the deposited mineralized matrix in the 100:0 and 95:5 constructs with long-term culture because the osteogenic differentiation of hMSCs was promoted by the HUVECs in a concentration-dependent manner. These results revealed that the amount of mineralized matrix increased and the cell constructs hardened following incorporation of HUVECs. Furthermore, it was demonstrated that the mineralized matrices were internally connected and formed a matured cancellous bone-like structure.

Our previous study reported that MSCs in the cell constructs formed mineralized matrices via the endochondral ossification process [17]. Because we observed that the hMSC/HUVEC construct also formed cartilage matrix, we postulated that hMSCs underwent cell differentiation, and then formed biomimetic bone tissues within the cell construct.

Subsequently, FT-IR analysis was carried out to confirm the formation of biomimetic bone matrices following incorporation of HUVECs. The spectrum patterns obtained in the present study revealed that the hMSC/HUVEC constructs formed bone matrices more analogous to living bone tissue than the hMSC-only constructs.

In this study, we investigated the impact of incorporating endothelial cells into the MSC construct during short- and long-term culture. Our results indicated that hMSCs and HUVECs worked synergistically in that i) HUVECs, which formed the luminal structure, promoted the survival and differentiation of the hMSCs, and ii) the hMSCs promoted the survival and reticulate structure formation of the HUVECs. In addition, it was demonstrated that biomimetic cancellous bone-like tissues were fabricated by osteogenic induction of hMSC/HUVEC constructs.

## Conclusions

We have demonstrated in this study that 3D cell constructs can be fabricated and maintained by incorporating HUVECs into hMSC constructs. In these hMSC/HUVEC constructs, the survival rate and osteogenic differentiation of hMSCs were promoted. Furthermore, HUVECs formed reticulate and luminal structures under the influence of growth factors secreted from the hMSCs. As a result of long-term osteogenic induction, cancellous bone-like tissues were formed within the hMSCs/HUVECs constructs.

The technology highlighted in this report, which enabled incorporation of the vein endothelial cells into the 3D cell constructs, would be valuable for development of the *in vitro* tissue engineering of various organs. In particular, the 3D biomimetic bone material fabricated in this study would be a promising approach as a novel biomaterial in bone regenerative medicine and tissue engineering.

## Supporting Information

S1 Fig Proliferation of hMSCs and HUVECs in osteogenic differentiation medium. (A) Light microscope images of hMSCs and HUVECs cultured in osteogenic differentiation medium at days 1 and 12. (B) Cell number was counted up to day 12 of culture.
(TIF)

S2 Fig Cartilage matrices deposited in hMSC 3D constructs were detected by alcian blue staining. Cartilage matrices (blue) were formed under the surface layer, highlighted as red staining, at day 50 of culture.
(TIF)
