# Supplementary figures and images for: Fabrication of Biomimetic Bone Tissue Using Mesenchymal Stem Cell-Derived Three-Dimensional Constructs Incorporating Endothelial Cells
Source: PLoS One. 2015 Jun 5;10(6):e0129266. doi: 10.1371/journal.pone.0129266 (PMC4457484; doi:10.1371/journal.pone.0129266)

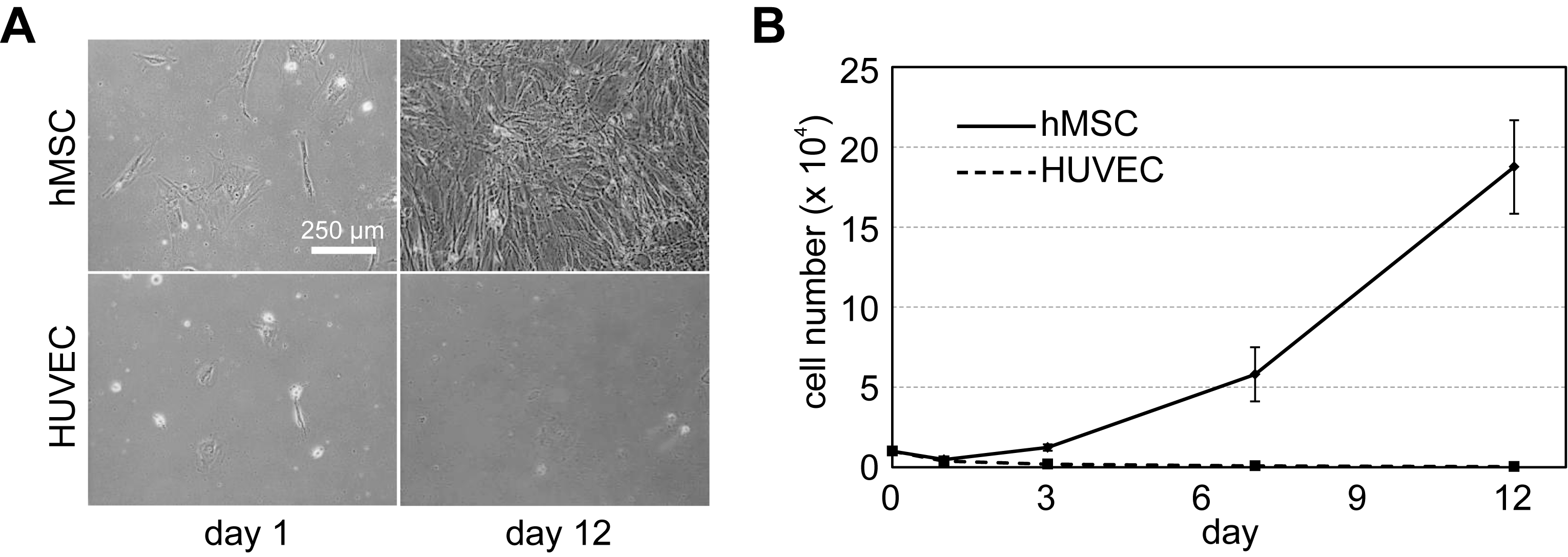

Supplement: S1 Fig — (A) Light microscope images of hMSCs and HUVECs cultured in osteogenic differentiation medium at days 1 and 12. (B) Cell number was counted up to day 12 of culture. (TIF) [file pone.0129266.s001.tif]

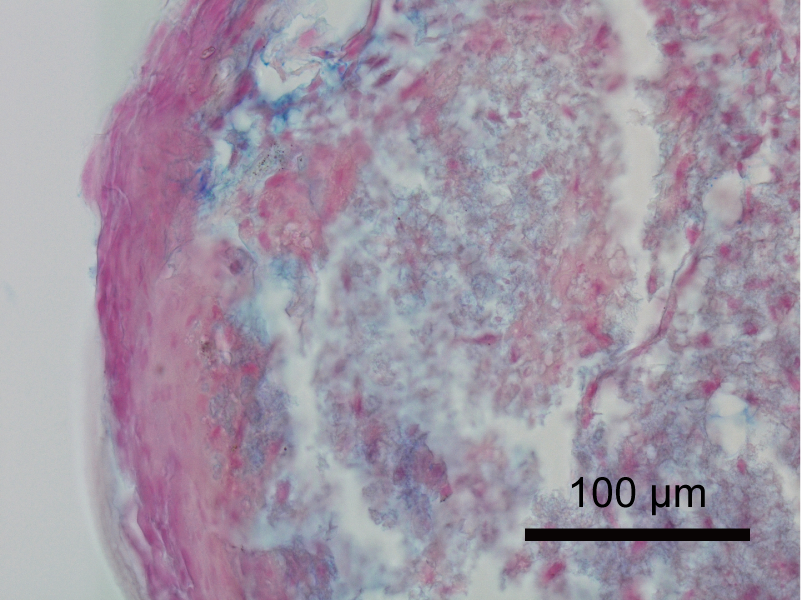

Supplement: S2 Fig — Cartilage matrices (blue) were formed under the surface layer, highlighted as red staining, at day 50 of culture. (TIF) [file pone.0129266.s002.tif]
